# Supplementary material for: Conflict reducing practices in evolution education are associated with increases in evolution acceptance in a large naturalistic study
Source: PLoS One. 2024 Dec 4;19(12):e0313490. doi: 10.1371/journal.pone.0313490 (PMC11616821; doi:10.1371/journal.pone.0313490)
Supplement: S1 Text — (DOCX) [file pone.0313490.s002.docx]

**S1 Text. Survey Items Used in Analyses and Final Outcomes**

**Acceptance of Human Evolution**

Please indicate whether you agree or disagree with the following statements, **based on your personal opinion.** (5-pt Likert-scale)

1. I think there is reliable evidence to support the theory that describes how humans were derived from ancestral primates.
2. I think that humans adapt, but they have not/do not evolve.*
3. I think that the physical structures of humans are too complex to have evolved.*
4. I think that humans and apes share an ancient ancestor.
5. I think that humans evolve.
6. I think that humans do not evolve; they can only change their behavior.*
7. I think the many characteristics that humans share with other primates (i.e., chimpanzees, gorillas) can be best explained by our sharing a common ancestor.
8. I think physical variations in humans (i.e. eye color, skin color) were derived from the same processes that produce variation in other groups of organisms.

* *Indicates reversed items.*

**Religious Denomination**

I most closely identify as:

- Buddhist
- Christian (for example, Catholic, Protestant, Orthodox, CJC-LDS, nondenominational)
- Hindu
- Jewish
- Muslim
- I don't identify with a religion (for example, atheist or agnostic)
- Option not available, please describe: ____
- Decline to state

**Religiosity**

Please indicate how much you agree or disagree with the following statements**:** (5-pt Likert-scale)

1. I attend religious services regularly (when they are available)
2. I believe in God
3. I consider myself a religious person
4. I consider myself a spiritual person

**Evolution Understanding**

This portion of the survey is meant to determine how much you understand about current evolutionary theory as proposed by scientists. Please answer the following questions based on your understanding of evolution. (True, False, I don’t know)

Please choose whether each statement is true or false based on your understanding of evolution:

1. Individuals don't evolve, species do.

2. Evolution is a progression towards more advanced species.

3. Mutations occur all the time.

4. Species evolve to be perfectly adapted to their environments.

5. In most groups of organisms, more offspring are born than survive.

6. Mutations can be passed down to the next generation.

7. More genetic variability makes a population more resistant to extinction.

8. Natural selection is the same thing as evolution.

9. The characteristics an organism acquires during their lifetime are often genetically passed down to their offspring.

10. Natural selection is the only cause of evolution.

11. The more recently species share a common ancestor, the more closely related they are.

12. Evolution means progression towards perfection.

13. Natural selection is a random process.

14. Natural selection means that only the smartest and physically strongest organisms survive.

**Examples of Compatibility Instruction**

Please answer the following questions regarding how you felt during the evolution instruction in this course (strongly disagree - strongly agree, 6 pt. scale)

1. I felt like the instructor helped me realize there are religious leaders who accept evolution.
2. I felt like the instructor helped me realize that there are scientists who accept evolution and are also religious.
3. I felt like the instructor helped me realize that for some religions their members can accept evolution.
4. I felt like the instructor helped me realize that there are religious people who accept evolution.

**Autonomy Instruction**

Please answer the following questions regarding how you felt during the evolution instruction in this course (strongly disagree - strongly agree, 6 pt. scale)

1. I felt like the instructor wasn’t trying to force me to accept evolution.
2. I felt like the instructor let me come to my own conclusion about evolution.
3. I felt like the instructor let me make up my own mind about evolution.

**Major**

Is your major in biology? (includes biomedical sciences, biology and society, conservation biology, genetics, neurobiology/physiology/behavior, microbiology, medical microbiology, molecular bioscience, neuroscience)

- Yes
- No
- I'm not sure (please describe): ________________________________________________
- Decline to state

**Gender**

- I most closely identify as:
- Woman
- Man
- Nonbinary
- Please describe your gender identity if the best option is not listed: __________
- Decline to state

**Race/ethnicity**

- Choose the race/ethnicity with which you most closely identify:
- American Indian, Native American, or Alaskan Native
- Asian (Middle Eastern, East Asian, Southeast Asian, South Asian, West Asian)
- Black or African American
- Hispanic or Latinx
- Native Hawaiian or Other Pacific Islander
- White
- Multiracial (please describe your multiple racial/ethnic identities) ________________________
- Option not available, please describe: _____________________________________
- Decline to state

**Quality Check**

In your honest opinion, should we use your survey responses in our study? 
If you did not pay attention to the questions or did not provide accurate answers, please mark "no" so we can remove your responses from our data set. There is no penalty. We only want to make sure we have the most accurate data possible.

- Yes
- No
