# Supplementary material for: Conflict reducing practices in evolution education are associated with increases in evolution acceptance in a large naturalistic study
Source: PLoS One. 2024 Dec 4;19(12):e0313490. doi: 10.1371/journal.pone.0313490 (PMC11616821; doi:10.1371/journal.pone.0313490)
Supplement: S2 Text — (DOCX) [file pone.0313490.s003.docx]

**S2 Text. Item fit statistics for partial credit Rasch models**

Values of 0.5- 1.5 are considered to indicate a good fit [1]. We examined mean square item fit statistics (MNSQ) to detect improbable response patterns. All four items measuring instructional practice of religious role models and two items measuring autonomy were within acceptable outfit and infit. We found infit values slightly above 1.5 for items “I felt like the instructor wasn’t trying to force me to accept evolution” and “I felt like the instructor thought that religious ideas are not valuable”, indicating slightly high response variability [1,2]. Item “I felt like the instructor helped me realize that there are scientists who accept evolution and are also religious”, item “I felt like the instructor helped me realize that for some religions their members can accept evolution”, and I felt like the instructor helped me realize that there are religious people who accept evolution” had outfit values slightly less than 0.5, indicating the response pattern was overly predictable [1,2] (See S2B Fig and Fig 1).

We also visualized item-person relationships using WrightMap and Item Characteristic Curve plot to identify potential gap between person measures and item measures and further explore the misfit items [3,4]. WrightMap showed that all religious negativity items corresponded to the uppermost range of person ability indicating that many items were very difficult to agree with (S3A Fig.). This plot also explains why the outfit values were less than 0.5 for some religion negativity items because there was low variance in students’ responses [5] (S3B Fig.). This result indicates that many students disagreed that religion negativity was occurring during instruction within this population. Using this measure within a population that experiences more religion negativity could be more appropriate in the future.

We also calculated person and item reliability using 1) expected a posteriori (EAP) separation index that estimates the internal consistency of items within different test takers with similar ability and 2) Weighted maximum likelihood estimation (WLE) that estimates person/performance consistency if test takers were given items with similar difficulty, and Cronbach’s alpha to see internal reliability of the items [2,6]

**References**

1. Wright BD, Linacre JM. Reasonable mean-square fit values. Rasch Measurement Transactions. 1994;8(3):370.

2. Bond TG, Fox CM. Applying the Rasch model: Fundamental measurement in the human sciences. Mahwah, NJ, US: Lawrence Erlbaum Associates Publishers; 2001. xxiv, 255 p. (Applying the Rasch model: Fundamental measurement in the human sciences).

3. Boone WJ. Rasch Analysis for Instrument Development: Why, When, and How? CBE Life Sci Educ. 2016;15(4):rm4.

4. Tornabene RE, Lavington E, Nehm RH. Testing validity inferences for Genetic Drift Inventory scores using Rasch modeling and item order analyses. Evo Edu Outreach [Internet]. 2018 Jul 17 [cited 2022 Jun 20];11(1):6. Available from: https://doi.org/10.1186/s12052-018-0082-x

5. Curtis DD. Person Misfit in Attitude Surveys: Influences, Impacts and Implications. International Education Journal [Internet]. 2004 [cited 2023 Jun 9];5(2):125–43. Available from: https://eric.ed.gov/?id=EJ903843

6. Sbeglia GC, Nehm RH. Do you see what I‐SEA? A Rasch analysis of the psychometric properties of the Inventory of Student Evolution Acceptance. Science Education [Internet]. 2019 Mar [cited 2022 Mar 2];103(2):287–316. Available from: https://onlinelibrary.wiley.com/doi/10.1002/sce.21494
