# Supplementary material for: Conflict reducing practices in evolution education are associated with increases in evolution acceptance in a large naturalistic study
Source: PLoS One. 2024 Dec 4;19(12):e0313490. doi: 10.1371/journal.pone.0313490 (PMC11616821; doi:10.1371/journal.pone.0313490)
Supplement: S1 Table — (DOCX) [file pone.0313490.s004.docx]

**S1 Table. Response Rate** **by Course**

| **Course** | **States** | **Semester** | **Class Size** | **Completed all surveys** | **Response rate for each course (%)** |
| --- | --- | --- | --- | --- | --- |
| 1 | Arizona | Fall2018 | 300 | 150 | 50% |
| 2 | Utah | Fall2018 | 300 | 106 | 35% |
| 3 | Utah | Fall2018 | 120 | 84 | 70% |
| 4 | New York | Fall2018 | 650 | 322 | 50% |
| 5 | California | Fall2018 | 312 | 273 | 88% |
| 6 | Hawaii | Fall2018 | 300 | 163 | 54% |
| 7 | Alabama | Fall2018 | *missing* | 179 | N/A |
| 8 | Wisconsin | Fall2018 | 50 | 44 | 88% |
| 9 | Arizona | Spring2020 | 430 | 199 | 46% |
| 10 | Arizona | Spring2020 | 268 | 57 | 21% |
| 11 | Arizona | Spring2020 | 155 | 55 | 35% |
| 12 | Arizona | Spring2020 | *missing* | 61 | N/A |
| 13 | Utah | Spring2020 | 80 | 34 | 43% |
| 14 | Florida | Spring2020 | 141 | 72 | 51% |
| 15 | Arizona | Spring2020 | *missing* | 24 | N/A |
| 16 | New York | Spring2020 | 360 | 58 | 16% |
| 17 | Texas | Spring2020 | 575 | 293 | 51% |
| 18 | Texas | Spring2020 | 190 | 69 | 36% |
| 19 | Texas | Spring2020 | 440 | 213 | 48% |
| 20 | Alabama | Spring2020 | 83 | 40 | 48% |
| 21 | Alabama | Spring2020 | 275 | 155 | 56% |
| 22 | Alabama | Spring2020 | 221 | 71 | 32% |
| 23 | North Carolina | Spring2020 | 300 | 99 | 33% |
| 24 | North Carolina | Spring2020 | 137 | 43 | 31% |
| 25 | South Carolina | Spring2020 | 67 | 52 | 78% |
| 26 | Florida | Spring2020 | 617 | 367 | 59% |
| 27 | Florida | Spring2020 | 198 | 52 | 26% |
| 28 | Arizona | Fall2020 | 141 | 56 | 40% |
| 29 | Arizona | Fall2020 | 179 | 83 | 46% |
| 30 | Utah | Fall2020 | 390 | 119 | 31% |
| 31 | New York | Fall2020 | 640 | 196 | 31% |
| 32 | Texas | Fall2020 | 365 | 265 | 73% |
| 33 | Texas | Fall2020 | 232 | 66 | 28% |
| 34 | Texas | Fall2020 | 232 | 93 | 40% |
| 35 | Alabama | Fall2020 | 142 | 59 | 42% |
| 36 | Utah | Fall2020 | 128 | 53 | 41% |
| 37 | Florida | Fall2020 | 450 | 126 | 28% |
| 38 | California | Fall 2020 | 1311 | 567 | 43% |
| 39 | Arizona | Spring2021 | 166 | 115 | 69% |
| 40 | Arizona | Spring2021 | 98 | 55 | 56% |
| 41 | Florida | Spring2021 | 174 | 97 | 56% |
| 42 | Michigan | Spring2021 | 194 | 90 | 46% |
| 43 | Michigan | Spring2021 | 196 | 89 | 45% |
| 44 | North Carolina | Spring2021 | 189 | 81 | 43% |
| 45 | North Carolina | Spring2021 | 260 | 108 | 42% |
| 46 | North Carolina | Spring2021 | 200 | 134 | 67% |
| 47 | Texas | Spring2021 | 237 | 69 | 29% |
| 48 | Texas | Spring2021 | 172 | 24 | 14% |
| 49 | Texas | Spring2021 | 221 | 88 | 40% |
| 50 | Alabama | Spring2021 | 148 | 73 | 49% |
| 51 | Alabama | Spring2021 | 97 | 25 | 26% |
| 52 | California | Spring2021 | 931 | 335 | 36% |
| 53 | Florida | Spring2021 | 491 | 182 | 37% |
| 54 | Minnesota | Spring2021 | 143 | 65 | 45% |
| 55 | Texas | Spring2021 | 291 | 71 | 24% |
|  |  |  |  | **6719** | **44%** |
