# Supplementary material for: Conflict reducing practices in evolution education are associated with increases in evolution acceptance in a large naturalistic study
Source: PLoS One. 2024 Dec 4;19(12):e0313490. doi: 10.1371/journal.pone.0313490 (PMC11616821; doi:10.1371/journal.pone.0313490)
Supplement: S2 Table — (DOCX) [file pone.0313490.s005.docx]

**S2 Table. Details of Rasch transformation of Likert response options**

|  | **Reliability** | | | **Item fit** | |
| --- | --- | --- | --- | --- | --- |
| **Item names** | **Cronbach’s alpha** | **EAP separation** | **WLE separation** | **Unweighted (Outfit)** | **Weighted (Infit)** |
| **Compatibility** | | | | | |
| rsrm1 | 0.966 | 0.946 | 0.947 | 1.086 | 1.234 |
| rsrm2 |  |  |  | 0.889 | 1.015 |
| rsrm3 |  |  |  | 0.775 | 0.898 |
| rsrm4 |  |  |  | 0.762 | 0.891 |
| **Autonomy** | | | | | |
| aut1 | 0.853 | 0.847 | 0.784 | 1.421 | 1.591 |
| aut2 |  |  |  | 0.724 | 0.873 |
| aut3 |  |  |  | 0.736 | 0.887 |
