# Supplementary material for: Conflict reducing practices in evolution education are associated with increases in evolution acceptance in a large naturalistic study
Source: PLoS One. 2024 Dec 4;19(12):e0313490. doi: 10.1371/journal.pone.0313490 (PMC11616821; doi:10.1371/journal.pone.0313490)
Supplement: S3 Table — Estimated marginal means with standard error from a linear mixed model for human evolution acceptance among undergraduate biology students with different religious affiliations. Results are averaged over the levels of gender, race, and biology major. (DOCX) [file pone.0313490.s006.docx]

**S3 Table. Estimated marginal means with standard error from a linear mixed model.**

| Human Evolution Acceptance | | | | |
| --- | --- | --- | --- | --- |
|  | **Compatibility** | | **Autonomy** | |
| Religion | **Mean** | **SE** | **Mean** | **SE** |
| No religion | 0.12 | 0.07 | 0.14 | 0.08 |
| Christian | -0.19 | 0.05 | -0.16 | 0.06 |
| Other | 0.06 | 0.07 | 0.05 | 0.07 |

Estimated marginal means with standard error from a linear mixed model for human evolution acceptance among undergraduate biology students with different religious affiliations. Results are averaged over the levels of gender, race, and biology major.
