# Supplementary figures and images for: Conflict reducing practices in evolution education are associated with increases in evolution acceptance in a large naturalistic study
Source: PLoS One. 2024 Dec 4;19(12):e0313490. doi: 10.1371/journal.pone.0313490 (PMC11616821; doi:10.1371/journal.pone.0313490)

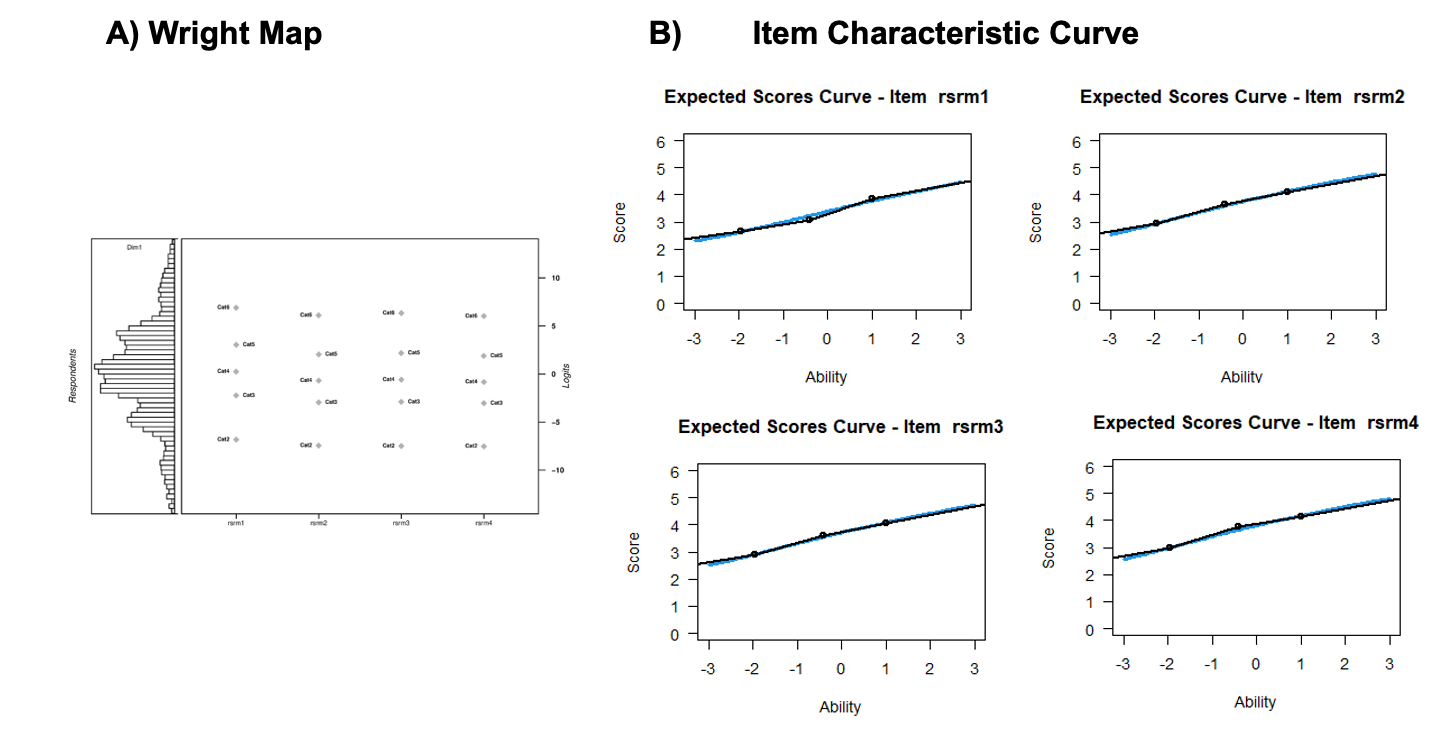

Supplement: S1 Fig — The histogram on the left represents the distribution of individuals’ “ability”, or test taker’ attributes. The score of individual ability was standardized in logits with 0 as the average and a higher value indicates a student agreed more compared to other students. The data point on the right represents item “difficulties”, or items’ attribute. The higher point indicates more “difficult” items or more disagreement. The higher point indicates more “difficult” items or more disagreement. For example, Cat6 is “strongly disagree” and Cat1 is “strongly agree”. (TIF) [file pone.0313490.s007.tif]

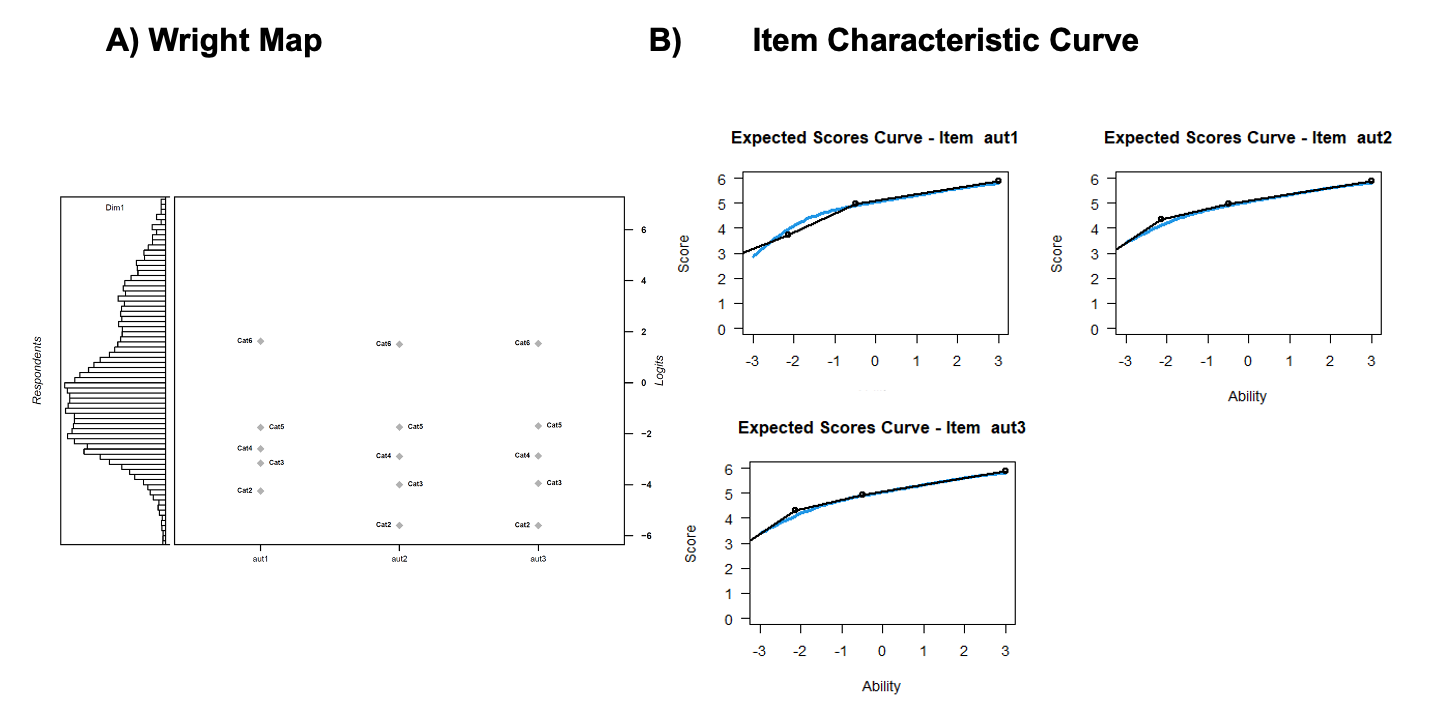

Supplement: S2 Fig — The histogram on the left represents the distribution of individuals’ “ability”, or test taker attributes. The score of individual ability was standardized in logits with 0 as the average and a higher value indicates a student agreed more compared to other students. The data point on the right represents item “difficulties”, or items’ attribute. The higher point indicates more “difficult” items or more disagreement. For example, Cat6 is “strongly disagree” and Cat1 is “strongly agree”. (TIF) [file pone.0313490.s008.tif]
