# Supplementary material for: Conflict reducing practices in evolution education are associated with increases in evolution acceptance in a large naturalistic study
Source: PLoS One. 2024 Dec 4;19(12):e0313490. doi: 10.1371/journal.pone.0313490 (PMC11616821; doi:10.1371/journal.pone.0313490)
Supplement: Attachment — Submitted filename: Response to reviewers.docx [file pone.0313490.s009.docx]

Dear monitoring editor and reviewers,

Thank you for your detailed and thoughtful review of our manuscript, "Conflict-reducing practices in evolution education are associated with increases in evolution acceptance: A large naturalistic study," for further consideration in PLOS ONE. We have carefully considered the feedback and believe we have significantly improved the manuscript.

First, we expanded the discussion to enhance the manuscript's potential for impact. We also expanded the description of the theoretical framework, particularly adding clarity to cultural competence as a frame. Additionally, we have made the limitations of our research more explicit, particularly regarding our inability to address the distinct experiences and perspectives of individual religious groups that are not Christian. We have also addressed the potential limitation of the generalizability of our sample. Another key revision involved improving the accuracy and clarity of our results, which reflected in the figures, figure captions, tables, and subheadings.

We have responded to each reviewer’s comments, as outlined by the editor, in more detail below. We hope these revisions bring the work closer to being suitable for publication

**PLOS ONE Style Requirements**

*We have ensured that our manuscript now meets the PLOS ONE style requirements, including file naming conventions. We have formatted the manuscript according to the provided PLOS ONE style templates.*

**Funding statement**

This study is funded by National Science Foundation #1818659. The funders had role in data collection.

**Supporting Information Files.** *We have included captions for our Supporting Information files at the end of the manuscript and updated the in-text citations to match accordingly.*

**Table and Figures:**

1. Table 1: The heading "Acceptance of evolution" may be misleading, as the percentages might not reflect this concept accurately.

*Thank you for your careful review. We agree that the heading "Acceptance of evolution" in Table 1 was not the correct heading and so we have deleted it.*

2. Figures 3 and 5:
   - Clarify the categories as "more than 1 SD above/below the mean" and use labels like "≤ -1 SD" or "≥ +1 SD" for clarity. *The categories are now clarified as "more than 1 SD above/below the mean," with labels like "≤ -1 SD" and "≥ +1 SD" for clarity.* (Lines 482, 486, 519, 524)
   - Resolve the inconsistency regarding the description of "students affiliated with no religion" by rephrasing the caption. *We have rephrased the caption to* “….*students who identify with no religious affiliation and students with religiosity measures more than 1 SD below the mean, which we have coded as having no religious affiliation” to resolve the inconsistency.* (Lines 485-487 and Lines 523-525)
   - Define what "mean" represents in the context of these figures to avoid confusion. *The figure caption was rephrased by changing "mean" to “average score for each course” and “average score within all samples” in the captions of Figures 1 and 3. (Line 463,464, 506, 507)*. *Additionally, "mean" was changed to “-1SD to +1SD” in the legends of Figures 3 and 5 to avoid confusion.*
   - Explain what the shaded areas in the plots signify, possibly the 95% CI for the linear model. *We also added an explanation of the shaded areas, indicating they represent the 95% confidence interval for the linear model.* (Lines 489 and 528)
   - Figure 4: Correct the dashed horizontal line, which should represent the "overall mean within all samples," and fix a copy-paste error in the R code. *Thank you for your careful review of our R code! We acknowledge that the dashed horizontal lines in Figure 4 represented the mean for the role models variable instead of the autonomy variable, and therefore, it was inaccurate. We have revised the R code (Line 501) and the figure to more accurately reflect the data.*

**Abstract:**

1. Add the background of the study at the beginning. *We have added the following to the abstract “Evolution is an important part of biology education, but many college biology students do not accept important components of evolution, like the evolution of humans. Practices that reduce perceived conflict between religion and evolution have been proposed to increase student evolution acceptance.”* (lines 21-24)
2. Include a statement about the potential transformative impact of the research on evolution education. *We have added the following “If instructors incorporate practices that emphasize one’s personal choice to accept or not accept evolution and compatibility, then these results suggest that students will leave their college biology classes accepting evolution more. Perhaps by using more conflict reducing practices, instructors can help increase evolution acceptance levels that have remained low in the United States for decades.”* (lines 40-44)
3. Specify the implications of the findings for evolution education practices. *We have added the following sentence, which underscores the potential for transformative impact as well as the implications for practices* “*If instructors incorporate practices that emphasize one’s personal choice to accept or not accept evolution and compatibility, then these results suggest that students will leave their college biology classes accepting evolution more. Perhaps by using more conflict reducing practices, instructors can help increase evolution acceptance levels that have remained low in the United States for decades.”* (lines 40-44)

**Introduction:**

1. Clearly state the research questions and objectives in a dedicated paragraph. *We have labeled a paragraph as “the current study” and more clearly articulated the research questions as “**(1) Are students’ perceptions of conflict reducing practices during their evolution instruction related to their evolution acceptance after instruction? (2) Is the effect of these conflict reducing practices different for Christian students, other religious students, and non-religious students?* (Lines 120-122; 137-141)
2. Include a brief discussion of the theoretical framework, particularly cultural competence in science education. *We added a paragraph on the theoretical framework of cultural competence and its origin and relevance to the current work* (Lines 95-110)

**Methods:**

1. Improve the clarity of the statistical analysis plan by using subheadings and explaining the rationale for each control variable. *Thank you for your suggestions. We have now separated the "Sample and Analyses" section into two distinct headings: "Participants" (Line 340) and "Analyses" (Line 410). Additionally, we have introduced subheadings such as "Main Effects…" (Line 414) "Interaction Effects…."(Line 437) and "Probing Interaction Effects" (Line 439) under the "Analyses" section. We have also included a brief rationale for each of our control variables to clarify their importance in the analysis.*
2. Address how the sampling methods ensure the representativeness of the sample and discuss the adequacy of the 44% response rate. *Thank you. We have added the following:* “*A 44% response rate is within the typical average for online surveys (Wu, Zhao, & Fils-Aime, 2022) and is particularly acceptable for a longitudinal study over an entire semester. The nature of the response rate for the survey is also influenced by various factors such as students’ interest in the topic, survey structure, communication methods, or incentives for participation (Saleh & Bista, 2017). Additionally, we implemented an honesty check at the end of the survey to assess whether students were paying attention and providing accurate responses. This step helped ensure the reliability of the data collected.”* (Lines 342-348)

**Results:**

1. Use subheadings to separate results for each research question or hypothesis. *We appreciate the suggestion, but we decided to have sub-headings in the result based on the instructional variables of autonomy and compatibility, for clarity. However, we incorporate the structure of discussion section based on research questions.*
2. Include effect sizes along with p-values to provide a sense of the magnitude of observed effects. *We used and have included regression coefficients that demonstrate effect size for fixed effect.*
3. Briefly mention the statistical methods used beneath the figures and tables. *Thank you for the suggestion. We have added to figure 3 and 5 that the results are based on linear mixed models (Lines 483 and 531). There were not statistical methods that we found could be added to the other tables and figures in the manuscript.*

**Discussion:**

1. Add more depth to the discussion section, particularly in interpreting the results. *Thank you for the opportunity to describe our results further in the discussion. We have added a new paragraph further analyzing students’ perceptions of the instruction called “student perceptions of conflict reducing practices in undergraduate biology education” (lines 536-564) and we have also broadened the discussion on why compatibility practices were only impactful for highly religious Christian students (lines 565-587).*
2. Address limitations more explicitly, especially the sample size for non-Christian religious groups. *This limitation is now more explicitly discussed in the revised manuscript in more detail and we discuss the limitations of the sample size for non-Christian religious students as well as the response rate and generalizability of the data to college biology students. We now say, “Further, it is possible that students in these classes we surveyed or those who chose to respond to the survey may not be representative of biology students more broadly. Although we cannot explore all ways the sample may be unique from the general population, we did compare key demographic characteristics of our sample with data from the National Science Foundation's National Center for Science and Engineering Statistics (NCSES, 2022) to assess how closely our sample aligns with the broader student population. Note that our sample consisted of students enrolled in introductory biology courses and only 54% of whom were biology majors, and therefore we compared with national data on degrees earned in science and engineering fields. The racial and ethnic composition of our sample is similar to national data. For instance, 51% of our sample identified as White (compared to 58% nationally), 18% identified as Asian (compared to 12% nationally), and 21% identified as PEER (Persons Excluded because of Ethnicity or Race), compared to 26% in the broader population. For gender composition, 70% of our sample identified as women, which is consistent with national trends. In 2020 (NCSES, 2022), women earned 66% of bachelor's degrees in science and engineering with a particularly high representation in fields like psychology (where women accounted for 79% of degrees earned). While we cannot claim full representativeness, these comparisons suggest that our sample broadly mirrors the diversity of the student population in relevant ways. It is also important to acknowledge the sample size limitations for students from non-Christian religious groups (e.g., Buddhist, Hindu, Jewish, Muslim), which led us to collapse these groups into a broader "Other-Religion Students" category. While this approach allowed us to maintain sufficient statistical power, it limits our ability to address the distinct experiences and perspectives of each individual religious group. Consequently, our discussion may not fully capture the unique nuances specific to students from smaller religious affiliations. (Lines 361–409463).*
3. Expand the discussion on why religious role models were effective only for Christian students, considering cultural and contextual factors. *We are grateful the reviewers asked us to expand this! We have added the following under the new heading “Are students’ perceptions of conflict reducing practices during their evolution instruction related to their evolution acceptance after instruction? In this study, when highly religious Christian students reported that instructors showed them examples of religious individuals who accept evolution, this was associated with increases in student evolution acceptance. This is in line with prior research that has documented the positive effect of religious role models who accept evolution on religious student evolution acceptance (15,18,20,48). However, in this study, we did not see a positive effect of these examples for religious students who were not Christian or Christian students who scored low to average on religiosity. This could be due to the religious affiliations of examples presented by instructors and the salience of the identity to students. Social identity theory would predict that students will respond more to examples of religious individuals that have affiliations closer to their own identities and when they themselves strongly associate with the identity. Thus, if instructors were presenting examples of religious leaders and biologists that were apparently Christian, this may have been more impactful for highly religious Christians compared to Christian students who did not report being as religious or compared to non-Christian students. Since we did not have observations of all instructors we cannot report the affiliations of examples instructors used, but of the courses we did observe that presented examples of compatibility, they were from Christian affiliations. Related, highly religious Christian students may be more likely than other religious students to even notice the representation of Christian role models who accept evolution.” (lines 565-584)*
4. Discuss the potential long-term effects of the conflict-reducing practices and their relevance to the field. *We have added the following sentence to the discussion “Data indicates that if instructors were to implement conflict reducing practices more regularly in undergraduate biology education, evolution acceptance rates will rise faster.” (lines 655-657)*
5. Consider adding a section on ethical considerations, including informed consent. *In the Materials and Methods section, we mentioned that the study was approved by Arizona State University’s Institutional Review Board (protocol #8191), and that both students and instructors provided informed consent prior to participation. To enhance clarity, we have added a subheading, "Ethical Considerations," where we expand on these points.* (lines 157-158)

**Title and Tone:**

1. Shorten the title to make it more concise. *We have shortened the title to “Conflict reducing practices in evolution education are associated with increases in evolution acceptance in a large naturalistic study”*
2. Add an introductory sentence in the abstract to address the research problem. *We have added the following to the abstract “Evolution is an important part of biology education, but many college biology students do not accept foundational components of evolution and may particularly struggle with accepting human evolution. Practices that reduce perceived conflict between religion and evolution have been proposed to increase student evolution acceptance.”* (lines 20-24)
3. Minimize the use of first-person pronouns to enhance the academic tone. *We have went through and minimized first person language where it did not compromise the clarity of the writing and ability of the audience to understand the specific actors involved in the research.*
4. Clarify the findings presented in the abstract to reflect all data sets collected. *We have added the following to the abstract to highlight the results of both conflict reducing practices “Linear mixed models showed that highly religious Christian students accepted evolution more when they perceived more compatibility practices. Further, students from all religious and non-religious affiliations accepted human evolution more after instruction when they perceived more autonomy practices. These results indicate that integrating examples of religion compatibility in evolution education will positively impact Christian students' views on evolution and that emphasizing students’ autonomy over their decision to accept evolution may be important for students more broadly.”* (Lines 34-39)

**General Suggestions:**

1. Ensure that the discussion includes more in-depth analysis of the findings rather than being dominated by comparisons with previous studies. *Again, thank you for the opportunity to describe our results further in the discussion. We have added a new paragraph further analyzing students’ perceptions of the instruction called “student perceptions of conflict reducing practices in undergraduate biology education) (lines 536) and we have also broadened the discussion on why compatibility practices were only impactful for highly religious Christian students (lines 564).*
2. Consider reorganizing the methodology section for better flow, particularly regarding the construct validation paragraph. *We reorganized the methodology section to place the paragraph on ‘construct validation’ after the paragraph on ‘expert and student reviews’ and before the paragraph on ‘class observation’* (Lines 220)

We did not repeat our responses above to respond to each individual reviewer comment, because the existing responses address all the individual comments below, but we do leave these aggregated individual comments below so the reviewers can easily find their comments and match how we addressed them above. We thank the reviewers for their help in making this work better and recognizing both the strengths and weaknesses of the work.

---------------------------------------------------------------------------------------------------------------------

**Reviewer 1**

Reviewer #1: This is a well-written, timely, and thoughtful research paper. The methodology, subject selection, and survey development all seem to be excellent.

There are a few small errors/inconsistencies that I happened to notice which should be corrected-- and I would ask that the authors please double-check all of the statistical analyses to ensure there aren't any additional errors. My concern is that a few sloppy (but minor) errors might indicate that something else has been overlooked.

Specific things that I happened to notice:

** In Table 1, there is a heading that says "Acceptance of evolution" which doesn't seem correct - I don't think those percentages indicate "acceptance of evolution."


** Figures 3 and 5 are not described completely or correctly. For instance:
- I think the categories should be described as "more that 1 SD above the mean" or "more than 1 SD below" instead of "1 SD above" or "1 SD below"? In the figure legend perhaps it would be clearer with a label like "≤ -1 SD" or "≥ +1 SD" ?

- The caption says "The red lines are students affiliated with no religion and have religiosity measures 1 SD below the mean." but in the text it states "students affiliated with no religion" in fact "may have checked the box for “Christian”, “Muslim”, “Jewish”, etc, we considered them non-religious because they did not believe this was a relevant or important part of their identities" So there seems to be some inconsistency there. Perhaps it would be better to state "The red lines are students with religiosity measures more than 1 SD below the mean, which we have coded as having no religious affiliation."

- Do the dashed lines that say "mean" represent the mean of the entire population of individuals coded in the specific subgroups (i.e. the mean of all students who identified as "Christian" or as "Other-Religion")? There are several uses of the term "mean" in the figure and caption and I can't always tell what is meant in each case.

- I couldn't find any description of what the shaded areas on these plots signifies— is it 95% CI for the linear model?


** In Figure 4 the dashed horizontal line should represent the "overall mean within all samples" but that obviously can't be correct. I found a simple copy-paste error in the R code (thank you SO MUCH for sharing your code! excellent.) - in line 379 in the file on GitHub it plots "course_stats_rsrm$mean" but should be plotting "course_stats_aut$mean"


I haven't tried to re-run the R code myself or repeat any of the analyses, but I would ask that the authors, in preparing a re-submission to correct these small points, please also ensure that there aren't other errors that I might not have noticed in my review of the manuscript. I think that the statistical analyses are appropriate and correct, but the small concerns identified above make me hesitant to give it my full confidence.

The discussion and survey of prior work in this area are thorough, and well-written. Excellent work!

**Reviewer #2: Dear Editor,**
Rahmi et al. have conducted a significant study on Student perceptions of conflict-reducing practices in evolution education. Their research, which is associated with increases in evolution acceptance, is a unique and valuable contribution to the field. With some major revisions, this study has the potential to become an even stronger piece of research.
Abstract:
The abstract provides a clear overview of the study's purpose, methods, and key findings. However, there are a few areas that could be improved.
1. Add the background of the study at the beginning of the abstract.
2. Consider adding a brief statement about the potential transformative impact of this research in the broader context of evolution education. This could inspire further research and innovation in the field.
3. The conclusion could be more specific about the implications of these findings for evolution education practices.
Introduction
1. The research questions are the backbone of the study and could be more clearly stated. Consider adding a paragraph that explicitly outlines the study's objectives and hypotheses, emphasizing their importance to the research process.
2. The introduction could benefit from a brief discussion of the theoretical framework guiding this research, particularly regarding the concept of cultural competence in science education.
Material methods
The statistical analysis plan is appropriate, but it could be more clearly presented. Consider using subheadings to separate the different analyses performed and provide a brief rationale for the inclusion of each control variable.

Results
The results are presented clearly and correspond well to the research questions. However, there are a few suggestions for improvement:
1. Consider using subheadings to separate the results for each research question or hypothesis.
2. It would be helpful to include effect sizes along with p-values to give readers a better sense of the magnitude of the observed effects.

3. Briefly mention the statistical methods used to derive key figures and results beneath the figures and tables.
Discussion
1. The discussion section provides a general interpretation of the results. While it lacks depth in explanation, it is evident that a significant amount of effort has been put into this section.
2. The analysis of differential effects across religious affiliations is somewhat limited by sample size for non-Christian religious groups. This limitation, along with others, could be addressed more explicitly in the limitations section, providing a more comprehensive understanding of the research's scope and potential areas for future investigation.
3. The discussion of why religious role models were only effective for Christian students could be expanded, perhaps considering cultural and contextual factors more deeply.
4. The potential long-term effects of these conflict-reducing practices are not addressed. A more detailed discussion of whether these changes in evolution acceptance persist over time would be valuable, as it would highlight the potential implications of the research and its relevance to the field.
5. Consider adding a brief section on ethical considerations, including how informed consent was obtained from participants.

Regards,

Reviewer #3: Title:
Your title is too long. It would be better if it could be shortened.

Abstract:
Please add an introductory sentence at the beginning of the abstract that addresses the research problem you are investigating.
You have used first-person pronouns excessively. To enhance the academic tone of the manuscript, minimize the use of first-person pronouns.
Based on the second and third sentences, it appears that you collected at least three sets of data. However, the fourth sentence only presents one finding.

Introduction:
The introduction is well-structured with a clear flow, allowing readers to follow the developed framework and the issues being reported effectively.
Although brief, the state-of-the-art and research gap related to the study’s topic are addressed in the fourth paragraph.

Methods:
The research methods are presented comprehensively, with sufficient detail and clarity, allowing readers to fully understand the design and steps of the study, from instrument development to the analysis of findings.
One minor suggestion for making the methodology section more systematic is to place the paragraph on construct validation for the survey instrument measuring Conflict Reducing Practices in Evolution Education (predictor variable) after the paragraph on expert and student reviews and before the paragraph on class observation.

Is a 44% response rate considered adequate?

Results:
The research findings are presented in a complete and systematic manner.

Discussion:
While the discussion addresses the findings, it lacks depth and breadth. The discussion is currently dominated by comparisons between your findings and those of previous studies, as well as suggestions for future research based on unanswered questions. These two components should not be reduced in the revised manuscript, but you should add more in-depth discussion of the findings you have obtained.

**Journal Requirements:**

When submitting your revision, we need you to address these additional requirements.

1. Please ensure that your manuscript meets PLOS ONE's style requirements, including those for file naming. The PLOS ONE style templates can be found at 
<https://journals.plos.org/plosone/s/file?id=wjVg/PLOSOne_formatting_sample_main_body.pdf> and 
<https://journals.plos.org/plosone/s/file?id=ba62/PLOSOne_formatting_sample_title_authors_affiliations.pdf>

2. Thank you for stating the following financial disclosure: 
"NSF Grant number 1818659"

Please state what role the funders took in the study.  If the funders had no role, please state: "The funders had no role in study design, data collection and analysis, decision to publish, or preparation of the manuscript." 
If this statement is not correct you must amend it as needed. 
Please include this amended Role of Funder statement in your cover letter; we will change the online submission form on your behalf.

3. Please expand the acronym “NSF” (as indicated in your financial disclosure) so that it states the name of your funders in full.
This information should be included in your cover letter; we will change the online submission form on your behalf.

4. Thank you for stating the following in the Acknowledgments Section of your manuscript: 
"We would like to acknowledge the instructors of biology courses willing to implement our survey and the thousands of students who were willing to complete the survey. This project was supported by the NSF Grant number 1818659."

We note that you have provided funding information that is not currently declared in your Funding Statement. However, funding information should not appear in the Acknowledgments section or other areas of your manuscript. We will only publish funding information present in the Funding Statement section of the online submission form. 
Please remove any funding-related text from the manuscript and let us know how you would like to update your Funding Statement. Currently, your Funding Statement reads as follows: 
"NSF Grant number 1818659"

Please include your amended statements within your cover letter; we will change the online submission form on your behalf.

5. Please include captions for your Supporting Information files at the end of your manuscript, and update any in-text citations to match accordingly. Please see our Supporting Information guidelines for more information: <http://journals.plos.org/plosone/s/supporting-information>.
